# Thermal stability of P-loaded Li-LSX zeolites for air separation

**DOI:** 10.1039/d6ra02978g

**Published:** 2026-07-02

**Authors:** Ming-Lei Gou, Dongyang Li, Ziwan Chen, Junhui Liu, Yongli Yang, Shuge Peng

**Affiliations:** a School of Chemistry and Chemical Engineering, Henan University of Science and Technology Luoyang Henan 471023 PR China mingleigou@haust.edu.cn +86 37964231914 +86 37964231914

## Abstract

To investigate the effects of phosphorus doping on the thermal stability and air separation of the low-silica X (LSX) zeolite, different amounts of phosphorus were loaded into the channels of a commercial Li^+^-exchanged LSX (Li-LSX) zeolite *via* wet impregnation. The pristine Li-LSX zeolite exhibited an exothermic peak above 500 °C, attributed to framework collapse, and its characteristic peaks of the FAU structure almost disappeared and transformed to the nepheline phase upon calcination at 600 °C. Meanwhile, due to the weaker electric field of Li^+^ by phosphorus, the weight loss of chemisorbed water in the phosphorus-modified zeolites exhibited a significant decrease with phosphorus addition, and the FAU structures were well preserved even after calcination at 600 °C. Notably, the Li-LSX zeolite modified with a low phosphorus loading (0.12 wt%) not only well preserved most elemental compositions and framework structure but also weakened the interactions of Li^+^ cations with water molecules, greatly improved thermal stability and exhibited a relatively higher N_2_ adsorption capacity and no significant differences in the O_2_ adsorption capacity with an N_2_/O_2_ selectivity coefficient of 8.39, which can fully meet the industrial requirements.

## Abstract 2

Phosphorus modified Li-LSX zeolites with excellent thermal stability and air separation performances.

## Graphical abstract

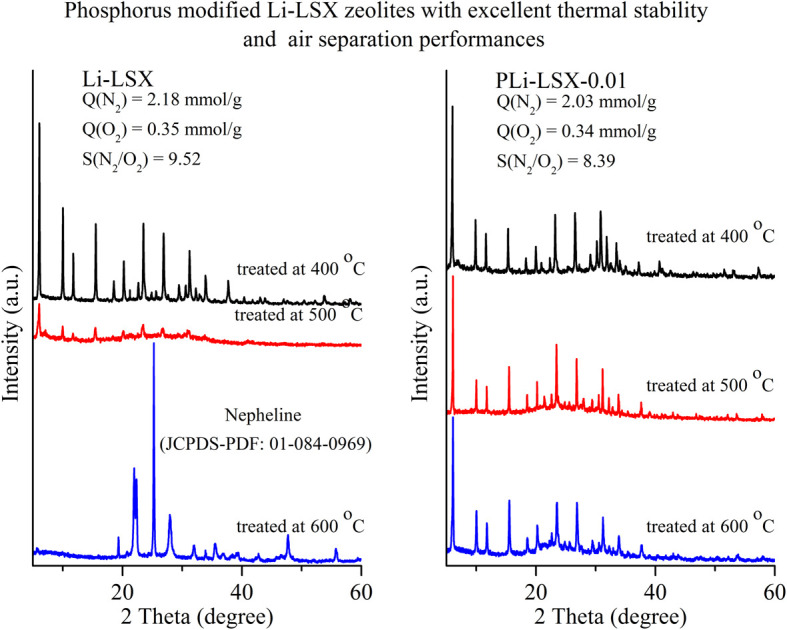

## 1. Introduction

Nitrogen (N_2_) and oxygen (O_2_), the primary constituents of the Earth's atmosphere (approximately 78 vol% N_2_ and 21 vol% O_2_), not only play important roles in the sustenance of life but also have extensive applications in industry and medicine.^1^ Ambient air separation provides an economic route to produce N_2_ and O_2_, both of which are non-polar molecules with nearly identical polarizability, molecular mass and size. Besides the cryogenic distillation of liquefied air for the high-volume and high-purity (>99.9%) production of N_2_, O_2_ and some inert gases based on their various boiling points at low temperatures, air separation at room temperature *via* pressure swing adsorption (PSA) or vacuum pressure swing adsorption (VPSA) is one of the main economic approaches for the low-to-medium scale production of both N_2_ and O_2_ with moderate purities (>90%). The success and challenges of these adsorption processes lie in the performance of adsorbents.^2,3^

Zeolites are ideal adsorbent candidates with the advantages of active extra-framework cationic sites, tunable composition and structure, as well as superior thermal and chemical stabilities.^4,5^ Low-silica zeolites, such as the low-silica X (LSX) zeolites based on the faujasite (FAU) framework with an Si/Al ratio of approximately 1.0, are the most effective adsorbents due to their highest aluminum content and the resulting maximum number of exchangeable cations.^6,7^ Indeed, due to the different quadrupole electric field gradients of N_2_ and O_2_, the origin of the air adsorption selectivity of zeolites is derived from their extra-framework cationic active sites.^8–10^ In 1989, Chao^11^ first demonstrated the excellent potential of Li^+^-exchanged LSX (Li-LSX) zeolites for air separation. To date, Li-LSX zeolites, as the preferred adsorbents for O_2_ production by air separation, have been extensively used in PSA/VPSA systems.^12,13^ However, the strong interactions between Li^+^ cations and water molecules generally lead to a decline in N_2_ adsorption capacity and N_2_/O_2_ selectivity. Consequently, feed air needs to be dried, and the Li-LSX zeolites require frequent dehydration *via* calcination at temperatures typically above 400 °C.^14^ Low-silica zeolites, especially the LSX zeolites, are more prone to thermal framework collapse and the subsequent amorphization or recrystallization of the zeolite framework, which further deteriorates their separation performance and shortens their service life in air separation applications.^15^

For developing better alternatives, extensive research has been performed on single-cation and mixed-cation exchanged LSX zeolites. Fan *et al.*^16^ revealed that the cationic radius and adsorbed water within the FAU framework had a remarkable influence on the thermal stability of LSX zeolites; for instance, the water adsorption capacity of Li-LSX was enhanced, but its thermal stability decreased compared with that of Na-LSX. Ca-LSX and Sr-LSX zeolites possess higher N_2_ and O_2_ adsorption capacity and N_2_/O_2_ separation factor, making them highly promising candidates to replace the Li-LSX zeolite.^17,18^ However, their thermal stabilities show no improvement; rather, they decrease with increasing alkaline earth cation size.^19^ Doping two or more types of cations into the Li-LSX zeolites has also been widely studied, such as alkaline earth cations (Ca^2+^,^20^ Mg^2+^,^21^ and Sr^2+^ (ref. 22)), transition metal cations (Ag^+^,^23,24^ Zn^2+^,^25^ and Cu^2+^ (ref. 26)) or rare-earth metal cations (Ce^3+^ (ref. 27)). The resulting mixed-cation LSX zeolites generally required a Li^+^ exchange level above 70 mol% to achieve satisfactory N_2_ adsorption capacity; unfortunately, their thermal stability has not been substantially improved.

Numerous researchers have confirmed that phosphorus modification can enhance the thermal and hydrothermal stabilities of zeolites because of the phosphorus species preferentially interacting with the tetrahedrally coordinated framework aluminum (TFAl) and suppressing dealumination.^28–30^ For example, different phosphorus precursors combined with various preparation strategies have been used to improve the hydrothermal stability of ZSM-5,^31,32^ β,^33^ Y^34,35^ and so on. But the effects of phosphorus modification on LSX zeolites remain insufficiently investigated in detail.

To investigate the effects of phosphorus modification on the thermal behavior and air separation of LSX zeolites, different amounts of phosphoric acid (H_3_PO_4_) were loaded into the channels of a commercial Li-LSX sample. Particular attention was paid to the migration and transformation of phosphorus species and their interactions with TFAl to enhance the thermal stability of the zeolite framework. These phosphorus-modified LSX samples were characterized by XRD, FT-IR spectroscopy, SEM, TG–DSC, nitrogen adsorption and ^27^Al, ^31^P MAS NMR, and the resulting effects on N_2_ and O_2_ adsorption capacity and separation performance were further investigated.

## 2. Experimental

### 2.1. Phosphorus modification of Li-LSX

Commercial Li-LSX zeolite (Si/Al ≈ 1.0, Li^+^ exchange degree = 99%) was supplied by Luoyang Jian Long Co., Ltd, China. Other reagents were commercially available and used without further purification. The phosphorus-modified samples were prepared by wet impregnation of Li-LSX with an aqueous solution containing the desired amount of H_3_PO_4_ at room temperature with a liquid-to-solid ratio of 4 ml g^−1^, and the slurry was mixed thoroughly and placed in open air for 24 h, followed by drying at 100 °C for 4 h. Prior to investigation, the samples were further treated at 400–600 °C for different durations. The as-prepared samples were denoted as PLi-LSX-*x-y-z*, where *x* indicated the concentration of H_3_PO_4_ (mol L^−1^), *y* indicated the calcination temperature (°C) and *z* indicated the calcination time (h).

### 2.2. Characterization

X-ray diffraction (XRD) patterns were recorded on a Bruker D8 Advance diffractometer with Cu Kα radiation (*λ* = 0.1541 nm) at a scanning speed of 5° min^−1^ in the range of 2*θ* = 5°–60°. Fourier transform infrared (FT-IR) spectroscopy was performed using a Nicolet 380 spectrometer by applying the KBr pellet technique. Scanning electron microscopy (SEM) coupled with an energy-dispersive spectrometer (EDS) was employed to examine the morphology and elemental composition of the samples on a Hitachi scanning electron microscope (FlexSEM1000).

The elemental contents in the samples were characterized by inductively coupled plasma atomic emission spectroscopy (ICP-AES, 715-ES, Varian), and the Si/Al molar ratio, Li/Al molar ratio, as well as phosphorus content (P wt%), were calculated.

The textural properties of the samples were analyzed by nitrogen adsorption at 77 K on a Quantachrome Autosorb-1 instrument. The total surface area (*S*_BET_) and total pore volume (*V*_total_) were calculated by the Brunauer–Emmett–Teller (BET) equation. The micropore surface area (*S*_micro_), external surface area (*S*_exter_) and micropore volume (*V*_micro_) were calculated by the *t*-plot method.

Magic-angle-spinning nuclear magnetic resonance (MAS NMR) spectroscopy was used to study the coordination environment of Al and P atoms within the zeolite framework. The ^27^Al and ^31^P MAS NMR spectra were measured on a Varian InfinityPlus-300 spectrometer with resonance frequencies of 78.13 and 121.37 MHz, respectively.

The thermal behavior of the samples was evaluated using thermogravimetry (TG) analysis and differential scanning calorimetry (DSC). The TG and DSC curves were obtained on a PerkinElmer Pyris 1 Instrument to determine the thermal behavior of the samples at a heating rate of 10° min^−1^ from 50 °C to 800 °C in an air atmosphere.

### 2.3. Measurement of air separation

For the practical application of PSA or VPSA air separation, the main components of the adsorption bed gas are N_2_, O_2_ and Ar.^36,37^ Due to the similar adsorption properties of O_2_ and Ar,^38^ the measurement of air separation performances of the samples focused on N_2_ and O_2_. The phosphorus-modified samples' nitrogen and oxygen adsorption isotherms were analyzed on a Micromeritics ASAP 2020 Sorptometer *via* a volumetric measurement technique. Prior to measurement, all the samples were degassed and dehydrated *in situ* to remove adsorbed moisture and gases. Subsequently, the isotherms of nitrogen and oxygen were tested at 25 °C under a pressure of 0–300 kPa, and the N_2_/O_2_ selectivity coefficient was calculated under 760 mmHg with the volume fraction ratio of N_2_ : O_2_ = 78 : 21 using pure-component adsorption isotherm data.

## 3. Results and discussion

### 3.1. Physical properties of the samples

**Fig. 1:**
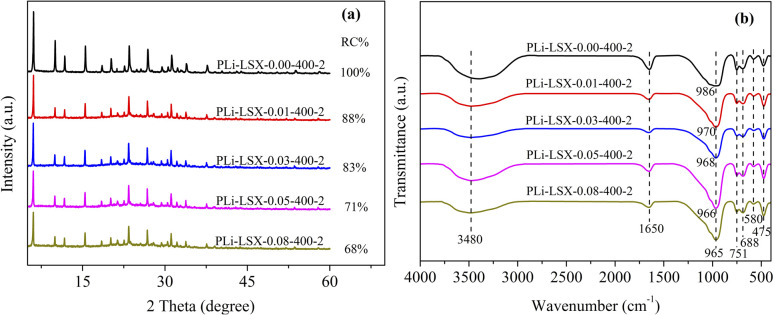
XRD patterns (a) and FT-IR spectra (b) of the phosphorus-modified Li-LSX zeolites.

Fig. 1 displays the XRD patterns and FT-IR spectra of the phosphorus-modified Li-LSX zeolites. As shown in Fig. 1(a), all the XRD patterns matched well with the typical diffraction patterns of the FAU structure, and no other peaks corresponding to phosphorus compounds were found, suggesting that phosphorus was highly dispersed on the surface of the samples. The relative crystallinity (RC) of the samples was calculated by comparing the total integrated areas of the peaks at 2*θ* = 6.1°, 10.0°, 15.4°, 23.3°, 26.6° and 30.9° with those of the pristine Li-LSX zeolite (*i.e.*, PLi-LSX-0.00-400-2). The phosphorus-modified samples showed a clear decrease in the intensity of the corresponding peaks with increasing phosphorus loading, *e.g.*, the RC values for PLi-LSX-0.01-400-2, PLi-LSX-0.03-400-2, PLi-LSX-0.05-400-2 and PLi-LSX-0.08-400-2 were calculated to be 88%, 83%, 71% and 68%, respectively. The incorporation of phosphorus into the zeolitic framework could result in dealumination and the formation of aluminophosphate species,^39,40^ especially for PLi-LSX-0.08-400-2, whose structure was irreversibly damaged, accompanied by partial amorphization of zeolite crystals. These structural variations can be further confirmed by SEM and ^27^Al MAS NMR analyses.

The FT-IR spectra of the samples are shown in Fig. 1(b). The bands at 3480 cm^−1^ and 1650 cm^−1^ are attributed to the stretching and bending vibrations of lattice water hydroxyl groups and Si–OH–Al groups.^16,18^ The decrease in the intensities of these peaks upon phosphorus modification clearly suggested that water absorption capacity was gradually reduced, which was also confirmed by TG analysis discussed in the following section. Besides, the bands at 1400–400 cm^−1^ are associated with the asymmetric or symmetric stretching vibration of the tetrahedral Si(Al)–O bond and double six-membered ring in the framework structures.^41^ Specifically, the Si(Al)–O bond was the basic characteristic bond of the LSX zeolite, so its vibration peak intensity around 965 cm^−1^ was the highest. The band around 965 cm^−1^ shifted to a lower wavenumber (from 986 cm^−1^ to 965 cm^−1^) with increasing phosphorus addition, confirming the incorporation of phosphorus atoms into the zeolite framework.^42^ Meanwhile, the band intensity became stronger as the phosphorus content increased, indicating more PO_4_ tetrahedra formed in the zeolite framework.

**Fig. 2:**
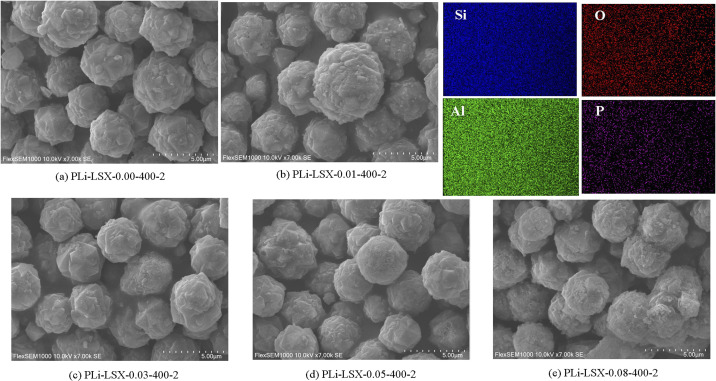
SEM (a–e) and elemental mapping images (upper right) of the phosphorus-modified Li-LSX zeolites.

The morphology and elemental analysis of the samples were assessed by SEM-EDS. As shown in Fig. 2, the particle morphology of the samples remains relatively intact with a size distribution of around 3–5 µm, suggesting that the phosphorus-modified samples maintained the FAU framework structure. However, with increasing phosphorus loading, the modified samples, especially PLi-LSX-0.08-400-2, presented more particle agglomeration and partial structural damage, which is in good agreement with the above XRD results. Taking PLi-LSX-0.01-400-2 as a representative sample, the colored elemental mapping images (upper right in Fig. 2) show that the main elements, such as Si, Al, O and P, are uniformly dispersed throughout the particles.

**Fig. 3:**
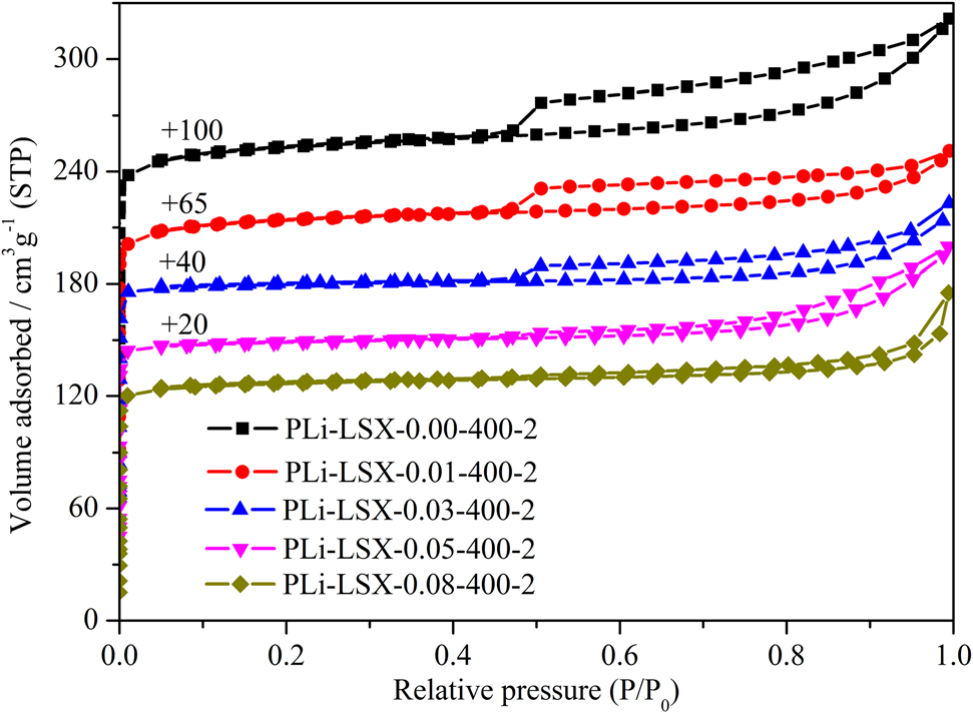
N_2_ adsorption–desorption isotherms of the phosphorus-modified Li-LSX zeolites.

Fig. 3 shows the N_2_ adsorption–desorption isotherms of the phosphorus-modified Li-LSX zeolites. All the samples present a sharp increase at *P*/*P*_0_ < 0.01, corresponding to typical I isotherms according to the IUPAC classification, indicating that most of the microporous structure was preserved after phosphorus modification. However, the phosphorus-modified samples have a lower adsorbed N_2_ quantity compared to Li-LSX. As shown in Table 1, the Li-LSX zeolite (*i.e.*, PLi-LSX-0.00-400-2) has the largest BET surface area (598.6 m^2^ g^−1^) and total pore volume (0.420 cm^3^ g^−1^), while both the BET surface area (592.1–457.2 m^2^ g^−1^) and total pore volume (0.408–0.196 cm^3^ g^−1^) of phosphorus-modified samples decrease gradually with phosphorus addition. This may be ascribed to dealumination and the formation of aluminophosphate species outside or near pore entrances, which blocked the zeolite channel openings.^43^ Furthermore, as compared to the phosphorus-modified samples, Li-LSX shows a more obvious hysteresis loop at *P*/*P*_0_ = 0.5–1.0, which is associated with the presence of inter-crystalline mesopores originating from the spontaneous assembly of crystalline particles. While the hysteresis loops of the phosphorus-modified samples are significantly diminished with phosphorus addition, and the same goes for their external surface area (20.3–9.6 m^2^ g^−1^), as shown in Table 1. Combined with the XRD and SEM results, these observations imply a partial loss of mesoporosity in the phosphorus-modified zeolites induced by particle agglomeration and structural damage upon phosphorus incorporation.

**Table 1:** Chemical composition and textural properties of the phosphorus-modified Li-LSX zeolites

Sample	Si/Al	Li/Al	P (wt%)	*S*_BET_ (m^2^ g^−1^)	*S*_micro_ (m^2^ g^−1^)	*S*_exter_ (m^2^ g^−1^)	*V*_total_ (cm^3^ g^−1^)	*V*_micro_ (cm^3^ g^−1^)
PLi-LSX-0.00-400-2	1.02	0.99	0	598.6	573.0	25.6	0.420	0.385
PLi-LSX-0.01-400-2	1.03	0.95	0.12	592.1	571.8	20.3	0.408	0.377
PLi-LSX-0.03-400-2	1.03	0.91	0.35	584.6	565.9	18.7	0.395	0.365
PLi-LSX-0.05-400-2	1.04	0.87	0.61	512.3	499.5	12.8	0.291	0.263
PLi-LSX-0.08-400-2	1.05	0.86	0.95	457.2	447.6	9.6	0.196	0.172

The Si/Al molar ratio, Li/Al molar ratio and phosphorus content (P wt%) determined by ICP-AES are listed in Table 1. The Si/Al ratios of the samples (1.02–1.05) slightly increased with increasing phosphorus loading, suggesting that a minor amount of aluminum was lost during phosphorus modification. According to the XRD results, the incorporation of phosphorus into the zeolitic framework could result in dealumination and the formation of aluminophosphate species, which reduces the number of charge-compensating extra-framework cationic sites (*i.e.*, Li^+^). Thereby, the Li/Al ratio of the phosphorus-modified zeolites declined from 0.95 to 0.86. Besides, the phosphorus loading (0.12–0.95 wt%) obtained *via* the wet impregnation method is close to the theoretically expected value.

**Fig. 4:**
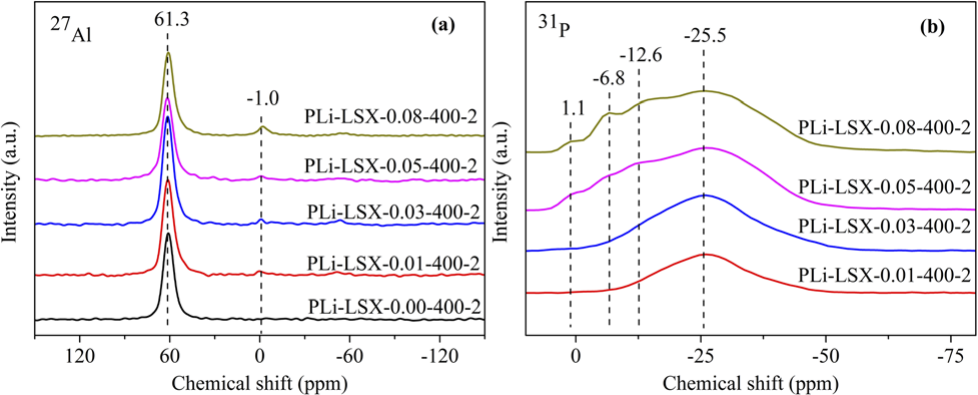
^27^Al (a) and ^31^P (b) MAS NMR spectra of the phosphorus-modified Li-LSX zeolites.

To further probe the coordination environment of atoms within the phosphorus-modified zeolites, ^27^Al and ^31^P MAS NMR experiments were carried out, with the results displayed in Fig. 4. It can be observed in Fig. 4(a) that each sample exhibits an intense peak at around 61.3 ppm, corresponding to the tetrahedrally coordinated framework aluminum (TFAl) species. After the introduction of phosphorus, a small peak at around −1.0 ppm, which is assigned to the octahedral extra-framework aluminum (EFAl) species, can be detected.^44^ With increasing phosphorus loading, the peak intensity of TFAl species presented a gradual decrease while that of EFAl species became a little stronger, indicating that the incorporation of phosphorus could result in slight dealumination of the Li-LSX zeolites, which is consistent with the above XRD results.

The chemical state of phosphorus species in the Li-LSX zeolites at various contents was observed by ^31^P MAS NMR spectra and displayed in Fig. 4(b). PLi-LSX-0.01-400-2 and PLi-LSX-0.03-400-2 gave a single broad resonance at around −25.5 ppm, which was caused by amorphous phosphates attached to aluminum (AlPO_4_).^45^ When a larger amount of phosphorus was impregnated on the Li-LSX zeolites, *i.e.*, PLi-LSX-0.05-400-2 and PLi-LSX-0.08-400-2, some minor new peaks at 1.1, −6.8 and −12.6 ppm were detected. The peaks at 1.1 and −6.8 ppm could be attributed to the formation of monomeric phosphates not connected to aluminum, whereas the peak at −12.6 ppm was related to terminal or intermediate groups in polyphosphates.^46^ It can be seen that most of the phosphorus existed as the AlPO_4_ species in the phosphorus-modified zeolites, along with a small amount of monomeric phosphates or polyphosphates with more phosphorus loading (*i.e.*, 0.61 wt% and 0.95 wt% as shown in Table 1). Phosphoric acid can coordinate with the TFAl species, inducing a small amount of Al to assume octahedral coordination, as evidenced by the ^27^Al MAS NMR results. Upon high-temperature activation or regeneration, P-TFAl (AlPO_4_) interfaces can retard dealumination and chemically prevent the irreversible collapse of the zeolite framework. Besides, phosphoric acid can distort the electronic environment of TFAl due to H-bonding between H atoms in H_3_PO_4_ and framework oxygen atoms or Brønsted acid sites and phosphate oxygen atoms, which would weaken the electric field of Li^+^ and reduce the polarization ability of Li-LSX zeolite toward water molecules.^34,45^ Further evidence for these phenomena will be presented in the subsequent section.

### 3.2. Thermal behavior of the samples

**Fig. 5:**
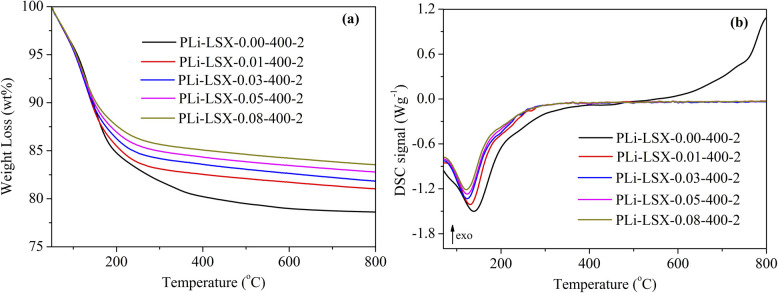
TG (a) and DSC (b) analysis of the phosphorus-modified Li-LSX zeolites.

To evaluate the thermal stability of the phosphorus-modified Li-LSX zeolites, TG and DSC profiles were first measured and are illustrated in Fig. 5. Prior to measurements, all the samples were exposed to 100% relative humidity at room temperature for 12 h. As shown in Fig. 5(a), almost all the water molecules can be desorbed within two temperature regions of below 150 °C and 150–400 °C, which are respectively related to the physisorbed water and chemisorbed water (such as crystal water, hydrogen-bonding water, or the water forming from hydroxyl and hydrogen atoms in two close positions).^47^ The weight loss of physisorbed water (approximately 10.0 wt%) was almost the same for Li-LSX and the phosphorus-modified zeolites. While the weight loss of chemisorbed water exhibited a significant decrease from 9.2 wt% to 5.2 wt% with phosphorus addition, which is likely due to the electric field of Li^+^ being gradually weakened by phosphorus, leading to a weaker ion polarization ability for water molecules.^48^

Furthermore, as shown in Fig. 5(b), due to relatively weaker bonding to water molecules after phosphorus modification, the DSC profiles showed endothermic peaks centered at 140 °C for Li-LSX zeolite (*i.e.*, PLi-LSX-0.00-400-2) and lower temperature (130–120 °C) for the phosphorus-modified zeolites, which can be attributed to the loss of absorbed water. Except for the phosphorus-modified zeolites, the Li-LSX zeolite exhibited an exothermic peak above 500 °C attributed to framework collapse, clearly indicating that the thermal stability of the Li-LSX zeolite is lower than that of the phosphorus-modified zeolites, which can be further confirmed by XRD and SEM analyses after being treated at a higher temperature.

**Fig. 6:**
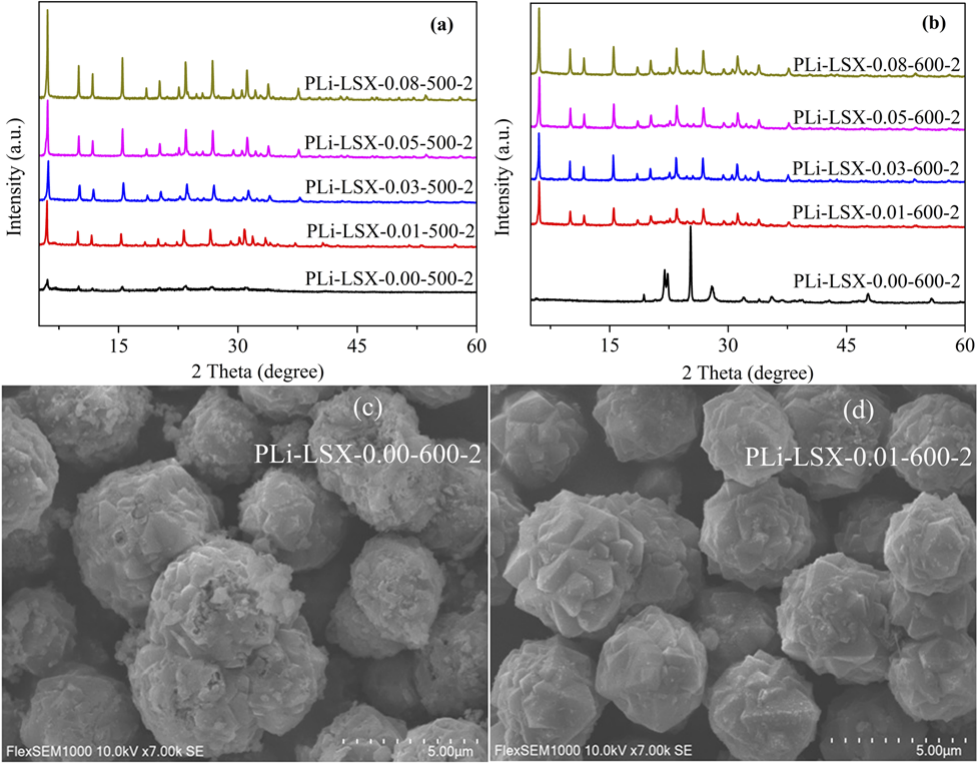
XRD patterns of the phosphorus-modified Li-LSX zeolites calcined at 500 °C (a) and 600 °C (b) as well as SEM images of Li-LSX zeolite (c) and phosphorus-modified zeolite (d) calcined at 600 °C.

The XRD patterns of the phosphorus-modified Li-LSX zeolites calcined at 500 °C and 600 °C are displayed in Fig. 6(a) and (b), respectively. Evidently, all the phosphorus-modified samples well retained the characteristic peaks related to the FAU structure after calcination at 500 °C and 600 °C for 2 h. In contrast, the pristine Li-LSX zeolite (*i.e.*, PLi-LSX-0.00-500-2) showed the nearly complete disappearance of FAU-related characteristic peaks upon calcination at 500 °C for 2 h. When the calcination temperature was further elevated to 600 °C, a phase transition from FAU to nepheline (JCPDS-PDF: 01-084-0969) occurred, as evidenced by the newly emerged diffraction peaks at 2*θ* = 21.9°, 22.3°, 25.3°, and 27.9°. SEM images of Li-LSX zeolite (*i.e.*, PLi-LSX-0.00-600-2) and phosphorus-modified zeolite (*i.e.*, PLi-LSX-0.01-600-2) calcined at 600 °C are shown in Fig. 6(c) and (d). Localized melting and framework collapse could be observed in the Li-LSX zeolite after calcination at 600 °C for 2 h, and simultaneously, the nepheline phase was detected by XRD. The phosphorus-modified zeolite, with just a small amount of phosphorus (0.12 wt% as shown in Table 1), could retain relatively intact spherical particles with a size distribution of around 3–5 µm even after calcination at 600 °C, consistent with the XRD and DSC profiles. Collectively, these results highlight the critical role of phosphorus modification in improving the thermal stability of the Li-LSX zeolites, which offers valuable insights for the development of low-silica zeolites.

### 3.3. Air separation performance

**Fig. 7:**
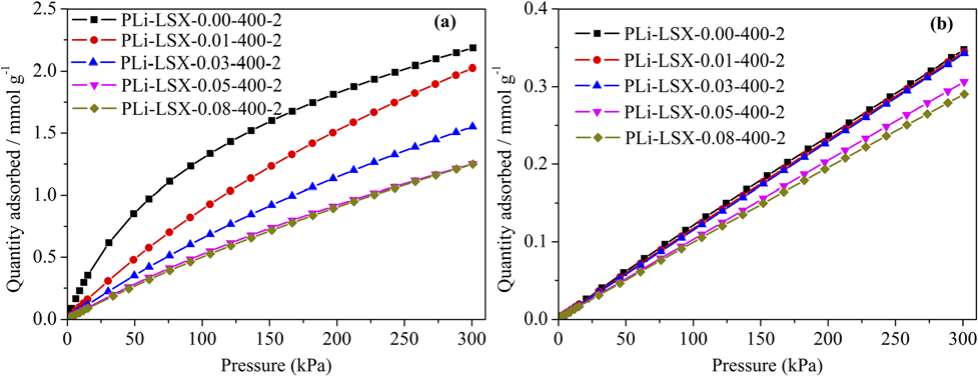
N_2_ (a) and O_2_ (b) adsorption isotherms of phosphorus-modified LSX zeolites at room temperature under a pressure of 0–300 kPa.

The N_2_ and O_2_ adsorption isotherms of phosphorus-modified LSX zeolites determined at room temperature (25 °C) under a pressure of 0–300 kPa are shown in Fig. 7. For evaluating the separation performance between N_2_ and O_2_, the Ideal Adsorbed Solution Theory (IAST) was utilized to calculate the selectivity coefficient (*S*(N_2_/O_2_)) of a mixed gas at 100 kPa with a volume fraction ratio of N_2_ : O_2_ = 79 : 21 based on the adsorption isotherm data for the pure components.^49^ The *S*(N_2_/O_2_) value was calculated by the following equation, and the corresponding results are summarized in Table 2.1
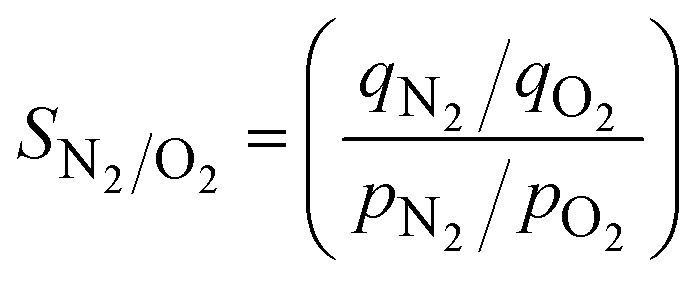
where *q*_N_2__ and *q*_O_2__ are the absolute loadings at partial pressures of *P*_N_2__ and *P*_O_2__.

**Table 2:** N_2_ and O_2_ separation performances of the phosphorus-modified Li-LSX zeolites

Sample	Quantity adsorbed (mmol g^−1^)		*S*(N_2_/O_2_)^a^
	*Q*(N_2_)	*Q*(N_2_)	
PLi-LSX-0.00-400-2	2.18	0.35	9.52
PLi-LSX-0.01-400-2	2.03	0.34	8.39
PLi-LSX-0.03-400-2	1.55	0.34	8.33
PLi-LSX-0.05-400-2	1.25	0.30	3.84
PLi-LSX-0.08-400-2	1.25	0.29	4.74

a IAST selectivity coefficients of N_2_/O_2_ at 100 kPa with the volume fraction ratio of N_2_ : O_2_ = 79 : 21.

For the Li-LSX zeolite (*i.e.*, PLi-LSX-0.00-400-2), the maximum adsorbed quantities of N_2_ and O_2_ at 300 kPa are 2.18 mmol g^−1^ and 0.35 mmol g^−1^, presenting the best N_2_/O_2_ selectivity coefficient (*S*(N_2_/O_2_) = 9.52) compared to the other phosphorus-modified samples. The above data and variation trend have agree well with the previous experimental results and simulations.^24,50^ When a small amount of phosphorus was impregnated on the Li-LSX zeolites, such as PLi-LSX-0.01-400-2 (0.12 wt%) and PLi-LSX-0.03-400-2 (0.35 wt%), the maximum adsorption quantity of N_2_ decreased to 2.03 mmol g^−1^ and 1.55 mmol g^−1^, but the O_2_ adsorption quantity (0.34 mmol g^−1^) remained almost unchanged. Thus, the N_2_/O_2_ selectivity coefficients gradually decreased to 8.39 and 8.33. However, when the amount of phosphorus impregnated on the Li-LSX zeolites increased to 0.61 wt% (PLi-LSX-0.05-400-2) and 0.95 wt% (PLi-LSX-0.08-400-2), the N_2_ adsorption quantity stayed around 1.25 mmol g^−1^, and the O_2_ adsorption quantity instead decreased to 0.30 mmol g^−1^ and 0.29 mmol g^−1^, resulting in a small increase in the N_2_/O_2_ selectivity coefficient from 3.84 to 4.74.

There are three different crystallographic sites (SI′, SII and SIII) in the Li-LSX zeolite.^51^ While only the Li^+^ cations in the site III (Li-III) near the four-membered ring inside the supercage can be available to interact with N_2_ molecules, and the other two sites (Li-I′ and Li-II), which are in the center of the six-membered rings located between sodalite cages and hexagonal prisms, as well as sodalite cages and supercages, are not accessible for effectively interacting with N_2_ and O_2_ molecules.^52^ From the above physical property analysis results (Section 3.1), the incorporation of phosphorus into the zeolitic framework could result in dealumination and the formation of aluminophosphate species, inducing a decline of the charge compensating extra-framework Li^+^ cationic sites. When a small amount of phosphorus was impregnated on the Li-LSX zeolites, the Li/Al ratios of PLi-LSX-0.01-400-2 and PLi-LSX-0.03-400-2 decreased to 0.95 and 0.91 compared to that of the Li-LSX zeolite (0.99), as shown in Table 1. Due to the larger permanent quadrupole moment of N_2_ than that of O_2_, stronger interactions occur between N_2_ and extra-framework Li^+^ cations, which play an important role in the N_2_ adsorption quantity and N_2_/O_2_ selectivity.^9^ Therefore, the N_2_ adsorption quantity of PLi-LSX-0.01-400-2 and PLi-LSX-0.03-400-2 gradually decreased, and the O_2_ adsorption quantity remained almost unchanged, accompanied by a slight decline in the N_2_/O_2_ selectivity coefficients. When a large amount of phosphorus was impregnated on the Li-LSX zeolites, the RC of PLi-LSX-0.05-400-2 and PLi-LSX-0.08-400-2 decreased to 71% and 68%, respectively, causing irreversible destruction, along with some amorphization of zeolite crystals according to the XRD analysis. As can be seen in Table 1, the Li/Al ratio of PLi-LSX-0.05-400-2 (Li/Al = 0.87) and PLi-LSX-0.08-400-2 (Li/Al = 0.86) had little difference, but their micropore surface area (*S*_micro_ = 499.5–447.6 m^2^ g^−1^) and micropore volume (*V*_micro_ = 0.263–0.172 cm^3^ g^−1^) obviously decreased, which would make a larger effect on N_2_ and O_2_ adsorption. Hence, the N_2_ adsorption quantity stayed around 1.25 mmol g^−1^, and the O_2_ adsorption quantity instead decreased to 0.30 mmol g^−1^ and 0.29 mmol g^−1^.

**Fig. 8:**
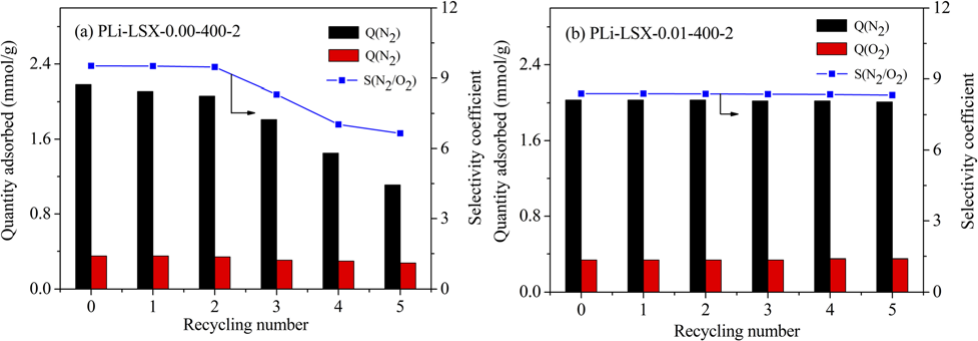
Performances of PLi-LSX-0.00-400-2 (a) and PLi-LSX-0.01-400-2 (b) for air separation in the recycling experiments.

It is well recognized that adsorbents are generally subjected to thousands of high-frequency cycling operations in practical industrial applications. To further compare the service lifetime and hydrothermal stability of phosphorus-modified LSX zeolites for air separation, the two samples (PLi-LSX-0.00-400-2 and PLi-LSX-0.01-400-2) were evaluated after pretreatment under harsh conditions: first exposed to 100% relative humidity at room temperature for 12 h, followed by calcination and dehydration at 400 °C for 2 h. Such harsh pretreatment can induce accelerated deactivation of adsorbent materials. The N_2_ and O_2_ adsorption isotherms were obtained at room temperature under a pressure of 0–300 kPa, and the maximum adsorbed quantity of N_2_ and O_2_ and N_2_/O_2_ selectivity coefficients are shown in Fig. 8. Both the adsorbed N_2_ quantity and the N_2_/O_2_ selectivity coefficients of Li-LSX zeolite (*i.e.*, PLi-LSX-0.00-400-2) obviously decreased after two cycles. While for the phosphorus-modified LSX zeolite (*i.e.*, PLi-LSX-0.01-400-2), no significant decrease in the adsorbed quantity of N_2_ and N_2_/O_2_ selectivity coefficients was noted in the recycling experiments. This suggested that the Li-LSX zeolite with a small amount of phosphorus loading (*i.e.*, 0.12 wt%), not only well preserved most of the elements and framework structure but also weakened the interactions of Li^+^ cations with water molecules and greatly improved the thermal stability, exhibiting a relatively larger adsorbed quantity of N_2_ with a slight decline of the N_2_/O_2_ selectivity coefficients. Although our results preliminarily confirm the stable performance of PLi-LSX-0.01-400-2 with no obvious degradation, the current evidence remains insufficient to support its industrial service life. This work only presents a preliminary evaluation of cycling stability.

## 4. Conclusions

Different amounts of phosphorus (0.12–0.95 wt%) were successfully loaded into the channels of the Li-LSX zeolites *via* wet impregnation. Small quantities of phosphorus loading (0.12–0.35 wt%) result in slight dealumination and changes of chemical composition and textural properties with the formation of only AlPO_4_ species. Meanwhile, with increasing phosphorus loading (0.61–0.95 wt%), the zeolitic framework is irreversibly destroyed, along with severe particle agglomeration and a considerable decrease in micropore surface area and micropore volume. The Li-LSX zeolite exhibits an exothermic peak above 500 °C, attributed to framework collapse, and the characteristic peaks of FAU structure almost disappeared and transformed to the nepheline phase upon calcination at 600 °C. Due to the electric field of Li^+^ being weakened by phosphorus, the weight loss of chemisorbed water in the phosphorus-modified zeolites exhibits a significant decrease with phosphorus addition, and the FAU structures are well preserved even after calcination at 600 °C.

The Li-LSX zeolite modified with a low phosphorus loading (0.12 wt%) not only well preserves most elemental compositions and the framework structure but also weakens the interactions of Li^+^ cations with water molecules and greatly improve thermal stability and exhibits a relatively larger adsorption amount of N_2_ (2.03 mmol g^−1^) and no significant differences in the O_2_ adsorption capacity (0.34 mmol g^−1^) with an N_2_/O_2_ selectivity coefficient of 8.39, which fully satisfies industrial application requirements.

## Author contributions

Ming-Lei Gou: formulation of overarching research goals and aims, writing – original draft preparation, reviewing and editing. Dongyang Li: experiments and other research outputs. Ziwan Chen: experiments and data analysis. Junhui Liu: investigation, methodology and formal analysis. Yongli Yang: investigation and data curation. Shuge Peng: supervision and resources.

## Conflicts of interest

There are no conflicts to declare.

## Supplementary Material

RA-016-D6RA02978G-s001

## Data Availability

Data for this article are available at https://www.scidb.cn/en/s/y2maUj. Supplementary information (SI): a detailed description of calculation of IAST selectivity. See DOI: https://doi.org/10.1039/d6ra02978g.
