# Supplementary material for: Thermal stability of P-loaded Li-LSX zeolites for air separation
Source: RSC Adv. 2026 Jul 2;16(34):32123–30. doi: 10.1039/d6ra02978g (PMC13325328; doi:10.1039/d6ra02978g)
Supplement: RA-016-D6RA02978G-s001 [file RA-016-D6RA02978G-s001.pdf]

Supplementary Information (SI) for RSC Advances.
This journal is © The Royal Society of Chemistry 2026

Supplementary data

Calculation of IAST Selectivity

The equilibrium between adsorbed phase and ideal gas phase can be expressed by equation (1)

$$Py_i = P_i^0 x_i \tag{1}$$

where y_i, x_i are mole fractions of component i in the gas and adsorbed phases, respectively. P (kPa) is the total pressure of the mixture, and P_i^0 (kPa) is the equilibrium gas phase pressure of pure component i corresponding to spreading pressure.

For a pure component i, the spreading pressure using Equations (2) was followed:

$$\frac{\pi_i A}{RT} = \int_0^{P_i^0} \frac{q_i}{P_i^0} dP_i^0 \tag{2}$$

where π_i is the spreading pressure of component i in the adsorbed phase, A is the specific surface area of the adsorbent, q_i is the absolute loading of component i.

Nitrogen adsorption can be fitted by the Langmuir model, while oxygen adsorption conforms to Henry's Law, so the equation (2) can be transformed into equation (3) and (4) for nitrogen and oxygen:

$$\frac{\pi_1 A}{RT} = \int_0^{P_1^0} \frac{b}{1 + aP_1^0} dP_1^0 = \frac{b}{a} ln(1 + aP_1^0)$$

(3)

$$\frac{\pi_2 A}{RT} = \int_0^{P_2^0} K dP_2^0 = KP_2^0 \tag{4}$$

where a, b are Langmuir constants, K is the Henry coefficient.

The separation performance parameter S predicted by IAST selectivity for the mixed fraction is

$$S_{i/j} = \left(\frac{q_i/q_j}{P_i/P_j}\right) \tag{5}$$

where q_i and q_j are the absolute loadings at partial pressure of P_i and P_j.

The iterative calculation results of IAST selectivity obtained via Python are presented in the Table S1.

Table S1. Iterative settlement results of ISAT selectivity (P_{total} = 100 kPa, y_{N2} = 0.79, y_{O2} = 0.21)

sample	P_{N2} (kPa)	P_{O2}(kpa)	q_{N2} (mmol/g)	q_{O2}(mmol/g)	S(N_2/O_2)
PLi-LSX-0.00-400-2	79.0	21.0	0.9887	0.0276	9.52
PLi-LSX-0.00-400-2	79.0	21.0	0.9692	0.0307	8.39

PLi-LSX-0.00-400-2	79.0	21.0	0.9690	0.0309	8.33
PLi-LSX-0.00-400-2	79.0	21.0	0.3776	0.0261	3.84
PLi-LSX-0.00-400-2	79.0	21.0	0.7257	0.0407	4.74
